# Prevalence of antimicrobial resistant *Escherichia coli* from patients with suspected urinary tract infection in primary care, Denmark

**DOI:** 10.1186/s12879-017-2785-y

**Published:** 2017-10-10

**Authors:** Gloria Córdoba, Anne Holm, Frank Hansen, Anette M. Hammerum, Lars Bjerrum

**Affiliations:** 1 The Research Unit for General Practice and Section of General Practice, Department of Public Health, University of Copenhagen; Øster Farimagsgade 5, 1014 Copenhagen, Denmark; 2 Department for Bacteria, Parasites and Fungi, Statens Serum Institut, Copenhagen, Denmark

**Keywords:** *E. coli*, Antibiotic resistance, Urinary tract infections

## Abstract

**Background:**

*Escherichia coli* is the most common pathogen causing Urinary Tract Infections (UTI). Data from the current National Surveillance program in Denmark (DANMAP) may not accurately represent the prevalence of resistant *E. coli* in primary care, because only urine samples from complicated cases may be forwarded to the microbiological departments at hospitals for diagnostic examination. The aim of this study was to assess the prevalence of resistant *E. coli* to the most commonly used antimicrobial agents in primary care in a consecutive sample of patients from general practice.

**Methods:**

Observational study carried out from December 2014 to December 2015. Thirty-nine general practices from The Capital Region of Denmark included adult patients with urinary tract symptoms and suspected UTI. All urine samples were sent to the central laboratory Statens Serum Institut (SSI). Significant bacteriuria was interpreted according to the European Urinalysis Standards. Susceptibility testing was performed and interpreted according to the European Committee on Antimicrobial Susceptibility Testing (EUCAST) standards.

**Results:**

From the 39 general practices 505 patients were recruited. Completed data were obtained from 485 (96%) patients. According to the European Urinalysis Standards, 261 (54%) patients had positive bacteriuria. The most common uropathogen in patients with uncomplicated (uUTI) and complicated (cUTI) urinary tract infection was *E. coli* 105 (69%) and 76 (70%), respectively. Eighty-two (45%) of 181 *E. coli* isolates were resistant to at least one of the tested antibiotics and 50 out of 82 isolates were resistant to two or more antimicrobial agents. The highest resistance-rate was found against ampicillin 34% (95% CI 24;42) in uUTI and 36% (24;46) in cUTI. There were no differences in the distribution of resistance between uncomplicated and complicated cases. The prevalence of resistance was similar to the one reported in DANMAP 2014.

**Conclusion:**

In *E. coli* from uUTI there is high resistance rates to antimicrobial agents commonly used in primary care. There was no difference in the distribution of resistant *E. coli* in suspected uUTI vs cUTI. In Denmark, data from the National Surveillance program DANMAP can guide the decision for choice of antibiotic in patients with suspected UTI seeking care in primary care.

**Trial registration:**

ClinicalTrials.gov NCT02249273.

**Electronic supplementary material:**

The online version of this article (10.1186/s12879-017-2785-y) contains supplementary material, which is available to authorized users.

## Background

Antimicrobial resistance is one of the most important threats to human health [1]. Multiple surveillance programs have been launched worldwide to monitor the spread of resistant strains in community acquired and nosocomial infections [2, 3].

Urinary tract infection (UTI) is the second most common bacterial infection managed in primary care [4, 5] and *Escherichia coli* is the most common pathogen causing UTI [6, 7]. *E. coli* resistant to antibiotics is on the rise, with great variation across regions [8, 9].

Experts recommend [10, 11] that choice of antibiotics in patients with suspected complicated UTI should be based on the results of a urine culture and susceptibility test, while the choice of antibiotics in patients with suspected uncomplicated UTI should be based on up-to-date surveillance data of patients from primary care. Thus, prospective surveillance of resistant patterns of uropathogens isolated from all patients attending primary care is crucial for guiding first and second line antibiotic selection.

Previous studies have suggested a systematic bias in surveillance data because uncomplicated UTIs (uUTI) are underrepresented, leading to an overestimation of resistance rates in primary care [12, 13]. This is problematic because general practitioners (GPs) need an accurate knowledge of the prevalence of resistance to the most commonly used antibiotics in primary care in order to make an appropriate treatment decision (i.e. choosing the right antibiotic).

In Denmark, the DANMAP programme is used for surveillance of antimicrobial consumption and antimicrobial resistance in bacteria from animals, food and humans [2]. DANMAP reports the prevalence of resistance for bacteria from clinical samples analyzed at the departments of clinical microbiology in Denmark. Part of the urine samples analyzed at the microbiology departments come from general practice.

The inferred prevalence of resistant strains in primary care may suffer from selection bias as GPs may predominantly send urine samples to culture in patients with complicated UTI or treatment failure.

In this paper, we report the results of a study aiming to assess the prevalence of resistant *E. coli* to the most commonly used antimicrobial agents in Denmark in patients with suspected (both complicated and uncomplicated) UTI seeking care at primary care level.

## Methods

### Study design

Prospective observational study carried out from December 2014 to December 2015.

### Participants

Five-hundred practices from The Capital Region of Denmark were randomly invited to participate. Thirty-nine practices accepted to consecutively recruit patients with the following characteristics: i.) inclusion criteria: Adult patients (i.e. > 18 years of age) seeking care in general practice during office hours with dysuria and/or urinary frequency as the main reason for consultation, and in which GPs suspected a UTI; ii.) exclusion criteria: a) Currently taking antibiotics, b) Inability to provide a urine sample, c) Inability to sign an informed consent, d) Previous participation in this study.

### Data collection

The day of the index consultation, all patients provided 10 mL of urine, which was sent to Statens Serum Institut (SSI). The urine sample was preserved in boric acid and sent by certified post the same day of the consultation.

### Culture and susceptibility testing at the reference laboratory

At SSI, the culture was analyzed by a medical laboratory scientist, who had no information about the clinical history of the patient. A positive culture was defined as growth of ≥10^3^ Colony Forming Unit per milliliter (CFU/mL) for *E. coli* according to the European Urinalysis Standards [14].

Aerobic urine culture was carried out with 1 μL on Blood agar plate and “Blue” agar plate (SSI Diagnostics; Hillerød, Denmark) and 100 μL on ESBL chromogenic culture media (Brilliance ESBL AGAR; Oxoid, UK).

ESBL plates were examined after one day of incubation and read according to the colour chart provided by the manufacturer. Phenotypic confirmation of ESBL production was performed by the Total ESBL Confirm Kit 98,014 (Rosco Diagnostics, Taastrup, Denmark).

Susceptibility testing was performed and interpreted according to EUCAST standards [15] on Mueller-Hinton agar plates using Neo-Sensitabs (Sulfamethoxazole, trimethoprim, ampicillin, amoxicillin-clavulanic acid, cefpodoxime, ciprofloxacin, nitrofurantoin and mecillinam (Rosco Diagnostics).

### Data analysis

We considered UTI as uncomplicated if the patient was a non-pregnant woman, under 65-year old without reported co-morbidity and assessed by the nurse or GP as not having an acute complicated cystitis or suspected pyelonephritis. In contrast, we considered UTI as complicated if the patient was a man, a pregnant woman, a woman 65-year or older or with a reported co-morbidity or assessed by the nurse or GP to have complicated cystitis or pyelonephritis.

The proportions of susceptible and resistant *E. coli* isolates were compared between uncomplicated and complicated UTI. Proportion of resistant *E. coli* isolates in our study was compared to the proportion of resistant *E. coli* isolates from primary healthcare from the National surveillance program DANMAP 2014. Significance of the differences between the independent samples were performed by using the Pearson’s Chi-Square test (alpha 5%; CI 95%) and Fisher exact test when appropriate. Descriptive analyses were performed using SAS software, Version 9.3 of the SAS System for Windows 7. Copyright (c) 2002–2010 by SAS Institute Inc., Cary, NC, USA.

## Results

### Baseline characteristics

**Fig. 1:**
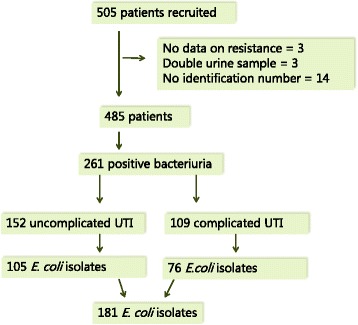
Flow chart of the study population

From the 39 practices, 505 patients were recruited. There was completed information for 485 (96%) of the patients, from which 261 (54%) had positive bacteriuria. Of the 261 cases, 152 (58%) were classified as uncomplicated UTI and 109 as complicated UTI. The most common uropathogen in uncomplicated and complicated cases was *E. coli* 105 (69%) and 76 (70%), respectively - Fig. 1.

### Antimicrobial resistance for *E. coli* isolates

**Fig. 2:**
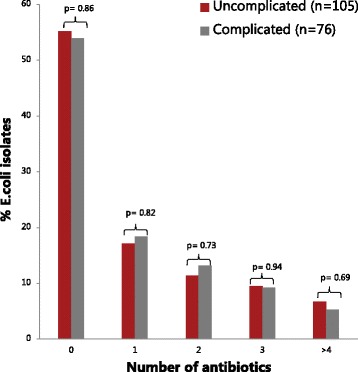
Distribution of susceptible and resistant *E. coli* isolates in uncomplicated and complicated cases

Eighty-two (45%) of the 181 *E. coli* isolates were resistant to at least one of the tested antimicrobial agents. Fifty (28%) of the 181 *E. coli* isolates were resistant to more than one antimicrobial agent. The distribution of resistance for the tested antimicrobial agents was not significantly different for the uncomplicated and complicated cases - Fig. 2 and Additional file 1: Table S1.

The highest resistance rates were found for ampicillin: 34% (95% CI 24;42) of the *E. coli* from uncomplicated cases and 36% (95% CI 24;46) of the *E. coli* from complicated cases. It was followed by sulfamethoxazole: 31%, (95% CI 21;39) of the *E. coli* from uncomplicated cases and 24% (95% CI 14;33) of the *E. coli* from complicated cases. Resistance to pivmecillinam (first line antibiotic in Denmark) was 1% (95% CI 0;5) in *E. coli* from uUTI and 9% (95% CI 2;15) from *E. coli* from cUTI. Resistance to third generation cephalosporins and clavulanate (i.e. ESBL-resistance test) was found in 6% (95% CI 1;10) of *E. coli* from uUTI and 3% (95% CI 0;9) of *E.coli* from cUTI. None of the tested *E. coli* isolates were resistant to nitrofurantoin – Table 1 and Additional file 1: Table S2.

**Table 1:** Resistance rates among *E. coli* isolates from patients seeking care in primary care

	Uncomplicated	Complicated
	*n* = 105	*n* = 76
Ampicillin	34% (24;42)	36% (24;46)
Sulfamethoxazole	31% (21;39)	24% (14;33)
Trimethoprim	23% (14;30)	17% (8;25)
Pivmecillinam	1% (0;5)	9% (2;15)
Ciprofloxacin	8% (2;12)	8%(1;14)
Nitrofurantoin	0	0
3rd gen. Cephalosporins + clavulanate^a^	6% (1;10)	3% (0;9)

^a^ESBL status tested with combinations of the third generation cephalosporins (Cefotaxime, Ceftazidime) and clavulanate

The differences between the resistance rates of *E. coli* isolates from the study population and DANMAP 2014 were lower than 10% across all antibiotics, for which comparison was available. No single difference was statistically significant. – Table 2 and Additional file 1: Table S2.

**Table 2:** Comparison of resistance rates for *E. coli* between the National Surveillance program DANMAP and our study

	DANMAP 2014	Our study^a^	*p*-value
Ampicillin	39%	34%	0.19
Sulfamethoxazole	32%	28%	0.2
Trimethoprim	N/A	20%	N/A
Pivmecillinam	5%	4%	0.74
Ciprofloxacin	9%	8%	0.5
Nitrofurantoin	N/A	0	N/A
3rd gen. Cephalosporins + clavulanate^b^	4%	4%	0.96

N/A no data from the National Surveillance program DANMAP 2014

^a^Uncomplicated and complicated cases

^b^ESBL- resistant *E. coli*

## Discussion

### Summary of main finding

This study shows that in uncomplicated cases there was high resistance to antibiotics commonly used in primary care in Denmark. There was no statistically and clinically significant difference in the distribution of resistant *E. coli*, in suspected uncomplicated vs complicated cases. Data from the National Surveillance program (DANMAP 2014) can be used to guide the selection of first and second line antibiotics to treat UTI in primary care in Denmark.

### Strengths and limitations

The pragmatic design of the study enabled the inclusion of a wide variety of patients seeking care in primary care due to urinary tract symptoms, thus uncomplicated and complicated cases were equally likely to be included in the study. It maximized generalizability to the patient population seeking care in primary care settings in Denmark.

Furthermore, all patients had a urine culture interpreted at the same reference laboratory. The laboratory technician had no access to clinical data. It minimized the risk for review bias and inter-observer variability.

The main limitation of our study is the small sample size resulting in lack of power to counteract the type II error (i.e. accepting the null hypothesis of lack of difference, when there is a difference between the groups). Nonetheless, the results of this study should be interpreted considering clinically relevant differences rather than statistically significant differences.

For example, in DANMAP 2014 the resistance rate of *E. coli* isolates to Sulfamethoxazole was 32%, while in our study it was 28%. The difference between estimates was not statistically significant and is not clinically relevant too. The Infectious Disease Society of America (IDSA) recommends that resistance rates above 20% is the threshold at which sulfamethoxazole is no longer recommended for empirical treatment [10].

Another example is the lower percentage of *E.coli* isolates from the uUTI group resistant to pivmecillinam in comparison to the cUTI group. Due to the small sample size, we cannot rule out that the difference in the point estimate was caused by chance.

Another limitation was that we relied on GPs judgment as part of the operationalization of the uncomplicated versus complicated variable. Thus, we cannot rule out that some patients may have been miss-classified as having uncomplicated UTI by their GP. Currently, there is no agreement about the criteria of classifying a patient as a uUTI or cUTI [16]. We chose to take into consideration the GPs’ assessment because it reflects more accurately the challenges for classifying patients as uUTI and cUTI during everyday practice.

### Comparison with other studies

The distribution of resistant *E. coli* in uncomplicated cases is similar to the distribution reported in other studies from Greece, Germany, Austria, Sweden, Portugal and the United Kingdom, in which ampicillin has the highest resistance and nitrofurantoin the lowest resistance rate [13, 17–20]. It confirms that *E. coli* resistant to antibiotics commonly used in primary care is an increasing problem, even in low prevalence settings such as the Danish context.

ESBL-producing *E. coli* was found in both uncomplicated and complicated cases seeking care at primary care level. Previous studies have already pointed out that ESBL- producing *E. coli* strains have the potential for spread beyond the hospital environment [21, 22]. Studies carried out in China [23] and Spain [24] have shown the constant increase of healthy carriers colonized with ESBL-producing *E. coli*. Thus, treating community-acquired urinary tract infections caused by ESBL-producing *E. coli* is a growing problem to be dealt with at primary care level as the therapeutic options are limited [25].

### Relevance

In Denmark, there are different guidelines made by different health authorities [26–28]. All guidelines agree on recommending pivmecillinam and sulfametizol as the first line options in patients with suspected uncomplicated UTI. All agree on pivmecillinam as first line options in patients with suspected complicated UTI, while only two suggest trimethroprim as an option too.

IDSA recommends that the selection of empirical antibiotics takes into consideration that resistance rates should not exceed 10% for fluoroquinolones and 20% for trimethoprim-sulfamethoxazole [10].

Based on the results of our study pivmecillinam is a good first option, while the routine use of sulfamethizol needs to be re-considered. In other countries, nitrofurantoin has started to gain importance as part of the first line antibiotics for the management of UTIs in primary care [10, 11]. A recent systematic reviews [29] about the efficacy and toxicity of short-term use (i.e. <14 days) of nitrofurantoin reported no differences in the rates for adverse events when compared to other antimicrobial agents and did not report cases of pulmonary fibrosis and hepatotoxicity.

Another alternative is Fosfomicyn [30]. It gives good bacterial coverage with low toxicity and limited effect in fecal flora, although it has low efficacy against *Staphylococcus saprophyticus*. A systematic review reported fewer adverse effect of fosfomicyn in pregnant women in comparison to other antibiotics used in primary care [31]. However, fosfomicyn is not licensed for use in Denmark; hence, it was not included for assessment in this study.

## Conclusion

Antimicrobial resistance is a rising problem that do not belong exclusively to patients attended in secondary care or complicated cases seen in primary care. In uncomplicated cases, there were high resistance rates to antibiotics commonly used in primary care. In Denmark, the National Surveillance program DANMAP can guide the decision for choice of antimicrobial agents in patients with suspected UTI seeking care in primary care.

## Additional file

Additional file 1: Table S1. Distribution of susceptible and resistant *E. coli* isolates in uncomplicated and complicated cases. **Table S2.** Number of resistant *E.coli* isolates in uncomplicated and complicated cases. (DOCX 19 kb)

## Abbreviations

cUTI: Complicated Urinary Tract Infection
DANMAP: Danish Programme for surveillance of antimicrobial consumption and resistance in bacteria from animals, food and humans
EUCAST: European Committee on Antimicrobial susceptibility testing
GPs: General Practitioners
IDSA: Infectious Diseases Society of America
SSI: Statens Serum Institute
UTI: Urinary Tract Infection
uUTI: Uncomplicated Urinary Tract Infection
