# Supplementary material for: Prevalence of antimicrobial resistant Escherichia coli from patients with suspected urinary tract infection in primary care, Denmark
Source: BMC Infect Dis. 2017 Oct 10;17:670. doi: 10.1186/s12879-017-2785-y (PMC5635483; doi:10.1186/s12879-017-2785-y)
Supplement: Additional file 1: Table S1. — Distribution of susceptible and resistant E. coli isolates in uncomplicated and complicated cases. Table S2. Number of resistant E.coli isolates in uncomplicated and complicated cases. (DOCX 19 kb) [file 12879_2017_2785_MOESM1_ESM.docx]

**Supplementary data:**

**Table 1. Distribution of susceptible and resistant *E. coli* isolates in uncomplicated and complicated cases**

|  | Uncomplicated  N = 105 | Complicated  N = 76 | p-value* | Total  N =181 |
| --- | --- | --- | --- | --- |
| Susceptible to all tested antibiotics | 58 (55.2) | 41(53.9) | 0.86 | 99 |
| Resistant to one of the tested antibiotics | 18 (17.1) | 14 (18.4) | 0.82 | 32 |
| Resistant to two of the tested antibiotics | 12 (11.4) | 10 (13.1) | 0.73 | 22 |
| Resistant to three of the tested antibiotics | 10 (9.5) | 7 (9.2) | 0.94 | 17 |
| Resistant to four or more of the tested antibiotics | 7 (6.6) | 4 (5.2) | 0.69 | 11 |

*Chi^2^ difference in proportions between complicated and uncomplicated cases

**Table 2. Number of resistant *E.coli* isolates in uncomplicated and complicated cases.**

|  | Ucomplicated  N = 105 | Complicated  N = 76 | Total  N =181 |
| --- | --- | --- | --- |
| Ampicillin | 35 | 27 | 62 |
| Sulfamethizol | 32 | 18 | 50 |
| Thrimethoprim | 24 | 13 | 37 |
| Mecillinam | 1 | 7 | 8 |
| Nitrofurantoin | 0 | 0 | 0 |
| Ciprofloxacin | 8 | 6 | 14 |
| 3rd gen. cephalosporins + clavulanate* | 6 | 1 | 7 |

*ESBL status tested with combinations of the third generation cephalosporins (Cefotaxime, Ceftazidime) and clavulanate.
